# Xenotransplantation Literature Update: July–December 2025

**DOI:** 10.1111/xen.70123

**Published:** 2026-03-30

**Authors:** Kasra Shirini, Joseph M. Ladowski, Niket Harsh, Raphael P. H. Meier

**Affiliations:** ^1^ Department of Surgery Johns Hopkins University School of Medicine Baltimore Maryland USA; ^2^ Department of Surgery Duke University School of Medicine Durham North Carolina USA; ^3^ Department of Surgery University of Maryland School of Medicine Baltimore Maryland USA

**Keywords:** clinical trials, genetic modifications, immunosuppression, kidney xenotransplantation, liver xenotransplantation, pig‐to‐human xenotransplantation

## Abstract

The second half of 2025 marked a significant transition for xenotransplantation, shifting further from experimental feasibility to early clinical translation. Prolonged physiologic support from genetically engineered porcine kidneys and livers in human recipients provided unprecedented insight into organ compatibility, rejection dynamics, and species‐specific physiology. Parallel advances in molecular profiling refined the understanding of innate and humoral immune injury, while innovations in donor‐pig engineering, immunomodulation, and biosafety frameworks strengthened translational readiness. Preclinical non‐human primate studies continued to inform clinical trial design, particularly regarding proteinuria, complement incompatibility, and novel xenoantigens. Alongside these scientific advances, growing attention to ethics, patient selection, and public trust highlighted the societal dimensions of clinical implementation. Collectively, these developments underscore the rapid maturation of xenotransplantation and define the scientific and regulatory foundations for ongoing first‐in‐human trials.

**Abbreviations**
AMR: Antibody‐mediated rejection
C1‐INH: C1 esterase inhibitor
CD: Cluster of differentiation
CTLA‐4: cytotoxic T‐lymphocyte–associated protein 4
ECM: Extracellular matrix
FDA: Food and Drug Administration
GTKO: α‐1,3‐galactosyltransferase gene knockout
IBMIR: Instant blood‐mediated inflammatory reaction
mTOR: Mechanistic target of rapamycin
NHP: Non‐human primate
pPBMC: Porcine peripheral blood mononuclear cell
PERV: Porcine endogenous retrovirus
pECM: Porcine pancreatic extracellular matrix
pRBC: Porcine red blood cell
POD: Postoperative day
RAAS: Renin–angiotensin–aldosterone system
RBC: Red blood cell
SLA: Swine leukocyte antigen
ST8Sia6: α‐2,8‐sialyltransferase 6
TNX‐1500: Anti‐CD154 monoclonal antibody

## 1 Introduction

Xenotransplantation entered a transformative phase in 2024–2025, driven by highly visible clinical progress and the initiation of formally authorized human studies. Multiple groups reported prolonged survival of genetically engineered pig‐to‐human kidney xenografts, reaching durations not previously achieved and providing unprecedented insights into graft function, immune interactions, and biosafety. In parallel, the first regulatory‐authorized clinical trials of kidney xenotransplantation were launched, with initial recipients demonstrating encouraging early clinical outcomes, while the disclosure of information on ongoing cases has significantly decreased, as required by regulatory standards. Collectively, these developments mark a clear transition of the field from preclinical experimentation and isolated compassionate‐use cases toward structured, hypothesis‐driven clinical investigation.

Key advances spanned donor‐pig genetic engineering, non‐human primate models, novel immunosuppressive and complement directed therapies, pathogen surveillance, advanced imaging and physiological monitoring, and ethical/societal considerations. This literature update summarized key peer‐reviewed xenotransplantation publications during this period, integrating emerging clinical data with mechanistic insights to contextualize the accelerating path toward safe, reproducible clinical application.

## 2 Progress in Clinic Xenotransplantation

Foundational histopathologic data have clarified the baseline appearance of porcine kidneys after transplantation into humans. Detailed biopsy assessment of 10‐gene–edited porcine kidneys in three human brain‐dead decedents demonstrated intact podocyte architecture, physiologically thin glomerular basement membranes, and incidental mesangial IgM/C3‐dominant immune‐complex deposits in pre‐implant biopsies, establishing a baseline morphology for clinical xenograft assessment [1].

Building on this baseline, subsequent clinical xenotransplantation clarified how rejection progresses in humans. A 61‐day α‐Gal knockout porcine kidney with a thymic autograft was transplanted into a nephrectomized brain‐dead decedent using clinical immunosuppression without CD40 blockade. By postoperative day (POD) 10, glomerular IgM/IgA deposition, early complement activation, and mesangiolysis occurred without proteinuria or functional decline. By POD33, antibody‐mediated rejection, characterized by rising creatinine and donor‐specific IgG, was fully reversed by plasma exchange, C3/C3b inhibition, and rATG. These findings underscore that antibody‐mediated injury can emerge despite substantial immunosuppression, but therapeutic temporizing measures exist [2]. A companion multi‐omics profiling delineated the immunological trajectory of this porcine xenograft. Schmauch et al. found an expansion of blood plasmablasts, NK cells, and dendritic cells occurring at POD10, followed by a dominant CXCL9^+^ macrophage infiltration and the development of combined antibody‐mediated rejection (AMR) and T‐cell–mediated rejection by POD49 [3]. Although strongly suspected based on previous reports, this study underscored the powerful innate immune pathways, particularly macrophage‐driven inflammation and complement activation, as pivotal in human immune xenograft injury. These cells will undoubtedly serve as high‐priority therapeutic targets in future work. Integration of comprehensive virus screening into OMICs analyses is essential to discriminate changes in gene expression induced by transplant rejection from those caused by viral infections [4].

Imaging modalities have also advanced our ability to detect early xenograft compromise. Ultrasound assessment of two xenokidney recipients demonstrated that elevated resistive indices and delayed enhancement on contrast imaging preceded biopsy‐confirmed rejection and improved following anti‐rejection therapy, suggesting that ultrasound could function as an early, non‐invasive surveillance tool [5]. The authors suggest that ultrasound biomarkers may serve as practical, bedside indicators of early xenograft compromise.

With these structural and functional insights as context, emerging clinical experience continues to refine our understanding of xenograft physiology. Data from the first‐in‐human genetically modified porcine kidney xenograft demonstrated that xenografts sustain essential renal functions, including stable urine output, electrolyte balance, and metabolic homeostasis, for up to 51 days. Additionally, these studies helped characterize the human–porcine physiologic differences such as urinary uric acid wasting causing profound hypouricemia, reduced renin–angiotensin–aldosterone system (RAAS) activation, persistent sodium avidity mediated by RAAS‐independent mechanisms necessitating diuretic therapy, mild hypocalcemia and hyperphosphatemia in the context of iatrogenic hypoparathyroidism, and manageable water balance with preserved responsiveness to human antidiuretic hormone [6]. Notably, the authors concluded that porcine kidneys can support complex human metabolic and endocrine functions, thereby establishing a clinical physiologic foundation for advancing clinical xenotransplantation trials.

Beyond the kidney, pig‐to‐human lung xenotransplantation also experienced significant progress: a lung xenograft in a brain‐dead recipient functioned reliably for 216 h without clear evidence of immediate hyperacute rejection. Although the graft ultimately failed after a few days, the hypothesis is that it succumbed to a combination of ischemia–reperfusion injury and humoral‐mediated rejection. The evidence for this is that severe edema occurred within 24 h, followed by consolidation consistent with primary graft dysfunction and CD68^+^ infiltration, and ultimately AMR by days 3–6 [7]. The authors concluded that while hyperacute rejection can be avoided, endothelial permeability and early antibody‐mediated injury remain significant barriers to lung xenotransplantation, as prior preclinical evidence had indicated.

Similarly, liver xenotransplantation studies further clarified the combined roles of coagulation and innate immunity. In a 10‐day pig‐to‐human decedent xenograft experiment, single‐cell and spatial transcriptomic profiling revealed progressive activation of circulating T cells and extensive infiltration of γδ T cells and exhausted T cells within the graft. Two monocyte populations with distinct functional signatures were identified: THBS1^+^ monocytes, enriched early after transplantation, interacted with platelets via THBS1–CD36 pathways and were predicted to modulate coagulation; and C1QC^+^ monocytes, which accumulated later and were associated with upregulation of CD274 (PD‐L1), suggesting promotion of local T‐cell exhaustion. Spatial analyses confirmed colocalization of these monocytes with zones of endothelial injury and fibrin deposition. Collectively, these findings indicate that cross‐talk between monocyte‐driven coagulation pathways and innate immune activation is a dominant mechanism limiting liver xenograft survival [8].

Zhang et al. reported the first auxiliary pig‐to‐human liver xenotransplantation using a 10‐gene–edited donor in a living recipient, in which the xenograft provided sustained metabolic, synthetic, and coagulation support for 38 days without hyperacute rejection. Despite early functional compatibility, xenotransplantation‐associated thrombotic microangiopathy ultimately limited graft durability, reinforcing the conclusion that coagulation dysregulation and innate immune injury remain central barriers to long‐term survival of liver xenografts [9].

Pharmacological and infectious‐disease management frameworks are also beginning to crystallize. A detailed report on the first living xenokidney recipient outlined strategies for donor pathogen screening, perioperative mitigation, and post‐transplant surveillance [10]. Pharmacokinetic analysis of daptomycin in a xenotransplant recipient revealed unexpectedly low AUC/MIC ratios despite clinical clearance of bacteremia [11]. The authors concluded that antimicrobial dosing standards may require adjustments tailored to xenograft‐specific conditions.

Finally, qualitative interviews with the three living xenotransplant recipients to date revealed themes of restored hope, improved daily function compared to dialysis, and the importance of trust and communication with clinical teams [12]. These perspectives underscore the human impact of xenotransplantation and reinforce the psychosocial feasibility of early clinical trials.

## 3 Preclinical Pig‐to‐NHP Organ Xenotransplantation

Preclinical cardiac xenotransplantation achieved an unprecedented advance with the development of orthotopic gene‐edited pig heart transplantation as a bridge to allotransplantation in infant‐sized baboons. In a pediatric pig‐to‐baboon model, Cleveland et al. performed 15 orthotopic cardiac xenotransplants using genetically engineered donor hearts and CD40/CD154‐based co‐stimulation blockade with rapamycin. More than half of the recipients survived beyond 1 month, with six surviving longer than 3 months. The longest‐surviving baboon maintained stable xenograft function for over 24 months. Importantly, prolonged xenograft exposure did not result in significant xeno‐ or allo‐sensitization, and selected recipients were successfully transitioned to cardiac allotransplantation demonstrating that orthotopic cardiac xenotransplantation represents a feasible and conceptually transformative bridging strategy for infants with end‐stage heart disease, establishing the most durable survival achieved to date in preclinical cardiac xenotransplantation and providing a strong rationale for future clinical translation [13].

Progress in cardiac xenotransplantation was achieved with the development of a standardized heterotopic abdominal pig‐to‐rhesus macaque model using GTKO donors. This platform optimized donor–recipient immunological and size matching, vascular anastomosis techniques, intraoperative procedures, and perioperative management. Its primary conclusion stressed the importance of this reproducible model for evaluating novel immunosuppressive regimens and investigating xenogeneic rejection mechanisms prior to clinical translation [14].

Additional preclinical insights into cardiac xenograft performance were gained through high‐resolution diffusion tensor imaging, which compared porcine and baboon myocardium. Notable differences in myocardial fiber orientation, particularly in E3‐angles across epicardial, midwall, and endocardial layers, suggest that intrinsic myocardial mechanical properties may contribute to xenograft overgrowth and maladaptive remodeling when exposed to the human hemodynamic environment. The investigators argue that further ex vivo and in vivo evaluation is necessary to clarify the functional implications of these structural differences for achieving long‐term cardiac xenograft stability [15].

Pig‐to‐baboon kidney xenotransplantation experiments identified nephrotic‐range proteinuria as a potential barrier to extended graft survival. Despite an aggressive immunosuppressive regimen incorporating escalated anti‐CD154 mAb, C1‐esterase inhibitor, B‐cell depletion, mTOR blockade, and IL‐6R inhibition 4 of 9 recipients developed severe proteinuria with histopathological evidence of glomerular thrombotic microangiopathy, focal segmental glomerulosclerosis, and/or transplant glomerulopathy. Notably, substantial urinary excretion of anti‐CD154 mAb was identified. The authors theorized that uncontrolled proteinuria could directly impair therapeutic antibody levels, potentially precipitating immune‐mediated graft injury [16].

Early rejection and heterogeneous outcomes remain an area of active investigation. One study by Jiang et al. found that unidentified porcine antigens may cause this early rejection phenomenon. The authors examined GTKO/β4GalNT2KO ± hCD55/hTBM donor pig kidneys that uniformly resulted in acute humoral xenograft rejection (AHXR) and graft loss within 30 days. Post‐porcine red blood cell (pRBC) absorption assays demonstrated persistent serum IgG/IgM binding and cytotoxicity to porcine peripheral blood mononuclear cells (pPBMCs), implicating either swine leukocyte antigen (SLA) or a novel neoantigen in complement activation and microvascular injury. [17]. While the existence of non‐Gal, non‐β4GalNT2 porcine antigens has been proposed previously, this work provides direct functional evidence linking such residual antigenic targets to early complement‐mediated vascular injury and uniform graft failure, strengthening the rationale for continued antigen discovery and expanded genetic modification strategies.

## 4 Xenograft Immunology and Rejection Mechanisms

Across human and preclinical xenotransplantation studies, convergent immunological patterns have become increasingly evident, such as the identification of innate immune activation as a consistent driver of early graft injury. For example, heart xenograft specimens assessed in brain‐dead human recipients through multi‐omic analysis demonstrated a significant endothelial cell activation accompanied by CD15^+^ neutrophil and CD68^+^ macrophage infiltration [18]. A similar innate‐immune signature was observed in a pig‐to‐human liver xenograft in a brain‐dead decedent, where 10‐day longitudinal single‐cell and spatial RNA sequencing revealed progressive peripheral T‐cell activation alongside extensive graft infiltration by γδT cells and exhausted T cells. Early post‐ transplantation, THBS1^+^ monocytes appeared to regulate coagulation through THBS1–CD36‐mediated interactions with platelets. Later, C1QC^+^ monocytes infiltrated the graft, potentially promoting T‐cell exhaustion via CD274 (PD‐L1) upregulation. [8].

Despite all the progress, complement–mediated cytotoxicity remains a major immunologic hurdle to long‐term xenograft success. Although eculizumab is able to suppress terminal complement pathway activation (C5b‐9 deposition) in normal human serum, in vitro studies found a diminished efficacy in rhesus serum. Conversely, C1‐INH yielded equivalent dose‐dependent suppression across species, albeit requiring elevated concentrations (≥ 10IU/ml) for substantial inhibition in monkeys. These findings underscore the need to understand the species‐selective pharmacodynamics when extrapolating preclinical outcomes to human clinical xenotransplantation [19].

## 5 Infectious Disease, Biosafety, and Virology

Robust biosafety frameworks remain essential as xenotransplantation enters early clinical trials. During this period, comprehensive protocols were established to standardize the detection of porcine viruses of most significant relevance to human recipients, including PCMV/PRV, HEV, PLHV, PERV, circoviruses, parvoviruses, and atypical pestiviruses. These PCR‐ and serology‐based workflows were designed for use in specialized virological diagnostic laboratories and aim to reduce the risk of donor‐derived viral transmission through highly sensitive and specific detection methods [20].

Preclinical virology studies provided further clarity regarding transmission risks. Although PLHV‐1, ‐2, and ‐3 were detected in nearly all donor pigs used in preclinical pig heart xenotransplantation studies, no evidence of PLHV transmission to the baboon recipients was observed, supporting the conclusion that PLHVs may pose lower cross‐species risk than previously feared [21]. In a comprehensive review, three additional types of in vitro co‐culture methods for detecting infectious human‐tropic PERV were analyzed, including their respective advantages and limitations [22]. In contrast, PERV remained a concern. A newly developed in vitro co‐culture method for porcine and human cells, using flowcytometry‐based cell sorting to isolate human cells specifically, demonstrated that PERV infection of human cells can occur within the first 24 h of contact if porcine cells exhibit high‐risk viral expression [23]. This work identified that even subtle differences in viral load or cell type can significantly alter transmission dynamics, reinforcing the need for selecting donor pigs with lower to absent risk of PERV infection. It is important to note that this is in vitro infection under permissive, high‐exposure conditions, not clinical disease, and it does not by itself establish clinical relevance.

Efforts to ensure the biosafety of xenogeneic biomaterials also continued to progress. Decellularized porcine liver scaffolds produced under optimized protocols were found to be effectively removed of all PERV‐specific DNA, RNA, and protein, substantiating their potential of using decellularized scaffolds of porcine origin for clinical applications without risk of PERV transmission [24]. Parallel vaccine development efforts demonstrated that neutralizing antibodies against the surface envelope (gp70) and transmembrane envelope (p15E) proteins of PERV can be generated in rats using an adjuvant type approved for human use. These findings provide proof of concept for the development of a protective vaccine to further enhance biosafety for xenotransplant recipients [25]. Cell culture experiments have shown that PCMV/PRV does not infect human cells, even when these cells have impaired antiviral defense mechanisms [26].

## 6 Islet and Cellular Xenotransplantation

Cell‐based xenotransplantation approaches advanced notably, with several studies identifying mechanisms to reduce early inflammatory injury, biomaterial‐mediated protection, and regulate humoral immunity.

A significant barrier to early islet xenograft survival remains the instant blood‐mediated inflammatory reaction (IBMIR), characterized by rapid activation of coagulation, complement, and inflammatory cascades upon blood exposure. Electrostatic conjugation of chitosan microparticles (CSMP) to islet surfaces enabled high‐density heparin loading (4.4 ng/IEQ) directly onto the islet exterior, providing localized anticoagulation without systemic exposure. This surface‐anchored strategy markedly attenuated coagulation and complement activation, reduced inflammatory cytokine release, and improved glycemic control and islet survival in vivo, supporting the feasibility of modular islet surface engineering approaches to mitigate IBMIR during the peri‐transplant period [27].

Parallel advances in islet preservation and microencapsulation focused on recreating biologically active microenvironments to enhance graft resilience. Microencapsulation using decellularized porcine pancreatic extracellular matrix (pECM) hydrogels provided a tissue‐specific niche that preserved islet structural integrity, enhanced glucose‐stimulated insulin secretion, and maintained expression of key β‐cell transcription factors, including PDX1 and MAFA, more effectively than conventional alginate microcapsules. pECM‐based microcapsules also conferred substantial resistance to hypoxic stress, significantly reducing islet apoptosis and promoting functional recovery following ischemic injury. In vivo transplantation studies in immunocompetent mice demonstrated favorable biocompatibility with minimal host immune activation, supporting the potential of pECM‐based hydrogels as immunoprotective and bioactive platforms for xenogeneic islet transplantation [28].

Beyond innate injury and biomaterial protection, emerging cellular and immunological studies have provided insight into the mechanisms controlling humoral xenoresponses. Single‐cell RNA sequencing analyses in rhesus monkey recipients of porcine islet xenografts demonstrated that belimumab‐containing, clinically applicable immunosuppressive regimens markedly reduced the frequency of both CXCR5^+^ and CXCR5^−^ atypical memory B‐cell subsets while broadly suppressing B‐cell activation signatures, including downregulation of CD69, CD83, and pathways associated with antibody‐secreting cell differentiation. These findings indicate that B‐cell activating factor (BAFF) blockade not only depletes potentially pathogenic atypical memory B cells but also induces functional impairment across the broader B‐cell compartment, highlighting its therapeutic potential for mitigating humoral rejection in clinical islet xenotransplantation [29].

Conversely, mechanistic studies evaluating long‐term graft maintenance underscored the continued necessity of sustained co‐stimulation blockade. Targeted interruption of CTLA‐4 signaling rapidly abrogated established graft acceptance in models using LEA29Y‐expressing porcine islet clusters, providing definitive evidence that ongoing co‐stimulation blockade remains essential for durable islet xenograft survival despite advances in biomaterial and humoral modulation strategies [30].

## 7 Ethical, Social, Psychological, and Regulatory Considerations

Rapid clinical progress in xenotransplantation has intensified reflection on its ethical, social, and regulatory dimensions. Foundational concerns continue to center on animal welfare, the risk of xenozoonotic infection, patient selection for first‐in‐human trials, and the imperative for transparent public engagement. Ethical analyses emphasized that the long‐term success of xenotransplantation depends not only on overcoming immunologic barriers but also on proactively addressing these societal obligations [31].

Considerations regarding vaccination also evolved, with comparative analysis suggesting that inactivated SARS‐CoV‐2 vaccines may stimulate broader polyclonal antibody responses, including anti‐pig cross‐reactivity, whereas mRNA‐based platforms may avoid these effects, underscoring the need for vaccine‐platform–specific guidance for xenotransplant candidates [32].

Surveys in Turkey revealed that religious appropriateness was the strongest independent predictor of acceptance among healthcare providers, transplant recipients, and dialysis patients, highlighting the importance of culturally sensitive communication strategies [33].

Among healthcare professionals, acceptance of kidney xenotransplantation reached 72.8% in a large survey of medical staff, with willingness to donate organs, prior awareness of xenotransplantation, and geographic region emerging as independent predictors, highlighting the need for targeted educational strategies within professional communities [34].

Finally, conceptual debates on chimerism and moral boundaries clarified that typical xenotransplantation does not meaningfully blur species identities. Empirical studies have shown that humans with animal organs are not perceived as morally ambiguous, and chimeric ethical concerns arise primarily when human cells contribute to the development of nonhuman brains. The overall conclusion was that xenotransplantation, as practiced clinically, does not provoke the kind of species‐boundary confusion that critics have hypothesized [35].

## 8 Patient Selection

A brief synthesis highlighted several system‐level aspects relevant to early patient selection, including the need for continued improvement in donor‐pig engineering and the establishment of standardized infectious disease management. It emphasized improved crossmatch approaches for identifying suitable candidates. These considerations were framed as practical components to support the safe and efficient design of clinical trials [36].

Advances in computational immunology further expanded tools for donor–recipient matching. An in silico “MHC Matchmaker” algorithm was introduced to compare MHC compatibility across pigs, NHPs, and humans at the amino‐acid level, achieving performance comparable to HLA Matchmaker used in allotransplantation. This platform may enhance the prediction of crossmatch outcomes, especially for highly sensitized patients, and lay the groundwork for the development of personalized xenografts in the future [37].

A data‐driven analysis of patient selection for early clinical xenotransplantation demonstrated that only a small fraction of waitlisted kidney patients would currently achieve survival equipoise unless xenograft survival reliably exceeds 2 years. Modeling approaches using Random Survival Forests, DeepSurv, and Cox proportional‐hazards methods further showed that achieving equipoise is highly sensitive to organ‐allocation policy, suggesting that priority adjustments for xenograft recipients could significantly expand eligibility while maintaining fairness across transplant centers [38].

## 9 Conclusion

The second half of 2025 was remarkable in the development of xenotransplantation, marked by prolonged human physiologic support from porcine organs, increasingly high‐resolution molecular maps of rejection pathways, significant advancements in the engineering of donor pigs, expanding biosafety infrastructures, and a deeper exploration of ethical, social, and regulatory issues. Together, these advances reveal the rapid pace at which this field is progressing from experimental proof‐of‐concept to formal early‐phase clinical translation.

Despite this momentum, several barriers remain central to future progress. These include early AMR driven by residual or newly identified xenoantigens, innate immune activation across organ systems, species‐specific complement incompatibilities, proteinuria‐driven loss of immunosuppressive agents in renal xenotransplantation, and heterogeneity in public understanding and acceptance. Progress in co‐stimulation blockade, genome engineering, tolerance induction, and organ‐preservation technologies, along with thoughtful, transparent engagement with patients and communities, will be key to surmounting these limitations.

As xenotransplantation enters its first regulated clinical trials, coordinated progress across molecular biology, immunology, infectious disease, pharmacology, ethics, and health systems science will set the path forward toward durable, safe, and equitable clinical applications. The findings emerging in this era represent both the scientific underpinnings and the social framework for the next generation of xenotransplantation research and practice.

## Conflicts of Interest

None of the authors have any conflict of interest to declare with regard to the content of this article.
